# Medical student elective during epidemics: a missed learning opportunity?

**DOI:** 10.1080/10872981.2020.1757901

**Published:** 2020-04-24

**Authors:** Shujhat Khan, Areeb Mian

**Affiliations:** Department of Medicine, Imperial College London, South Kensington, London

With the novel coronavirus being declared a global health emergency by the World Health Organisation, hospitals and medical sites in afflicted countries are hotspots for the virus to infect and replicate. In light of such news, many medical students have been refused permission to pursue an elective in such regions and neighbouring countries, by their respective universities. There are obvious reasons for this, with student safety and risk of viral dissemination being major concerns for universities. However, this has created an interesting debate amongst medical students. What should be the appropriate advice for traveling to epidemic countries not only during the current coronavirus outbreak but also in future epidemics?

The value for appreciating global health disease is clearly needed and is actively encouraged [1]. Despite the obvious dangers, first-hand exposure to rare, albeit highly hazardous diseases will present a unique learning opportunity and may ignite an interest in global health. However, as is often the case in underresourced areas, students may find themselves pressured to provide care beyond their role [2]. By having greater independence, students may have to diagnose and manage patients without strict clinical supervision. It is important to remember that medical students are not qualified doctors and it would not only be unethical but illegal to pretend to be a doctor as a student in most countries. However, in situations where there is a dearth of suitable staff, stretched resources and language barriers can create situations where patients may not realise that students are treating them, and indeed many would simply be happy to receive medical care. On the other hand, through utilitarian lenses, it can be argued that medical students should use their knowledge and capability for those in need [3], with an added side effect of relieving intense pressure from understaffed hospitals.

Advice on medical electives is constantly evolving and the provision of appropriate training has allowed students to embark on clinical rotations to areas that were once prohibited [4]. It should be noted however that medical students can offer so much more, from actively engaging in combatting viral misinformation to providing telemedicine for underappreciated illnesses that manifest as a consequence of mass infection [5]. As such, in the face of rapidly evolving global disasters, medical schools should be malleable in their strive for better learning experiences and should engage healthcare authorities whilst recognising their priority to student and public safety.
